# Dementia Friends in Incarcerated Settings: Development, Implementation, Evaluation, and Expansion

**DOI:** 10.1093/geroni/igaf122.1035

**Published:** 2025-12-31

**Authors:** Jessica Bibbo, Sarah Nicolay, Marty Williman, Bonnie Burman, Elizabeth Kinzig, Salli Bollin, Susan Hazlett, Lyndi Wyrostek

**Affiliations:** Benjamin Rose Institute on Aging, Cleveland, Ohio, United States; Benjamin Rose, Cleveland, Ohio, United States; Ohio Council for Cognitive Health, New Albany, Ohio, United States; Ohio Council for Cognitive Health, New Albnay, Ohio, United States; Ohio Council for Cognitive Health, New Albany, Ohio, United States; Ohio Council for Cognitive Health, New Albany, Ohio, United States; Summa Health System, Akron, Ohio, United States; MemoryLane Care Services, Toledo, Ohio, United States

## Abstract

In 2021, the HRSA-sponsored Geriatric Workforce Enhancement Program, Ohio Council for Cognitive Health, Benjamin Rose Institute on Aging, and the Ohio Department of Rehabilitation and Correction (ODRC) worked together to develop Dementia Friends for Incarcerated Settings to impact the lives of those living with dementia within the correctional setting. This pilot targeted ODRC staff and furthered the development of this Dementia Friends programming. The evaluation measured staff knowledge/attitudes about dementia, as well as uncovering the experiences and needs of staff within the ODRC. This paper will present results from the online staff training finalized in January 2023, including the optional embedded pre- and post-session surveys. The results (N = 743, n = 269 completed both pre and post, n = 213 post only) underscore the need, usefulness and applicability of dementia education throughout the ODRC system, indicating that teaching effective communication techniques within a correctional facility are helpful for those working within such settings. Further, the quantitative and qualitative results indicated benefits on both a personal and professional level. In 2023, work began on creating a Dementia Friends session for people who reside in incarcerated settings in Ohio. In January of 2025, an elearning program was launched nationally through Edovo, their first program focused on memory loss and dementia. Statistics on the usage and completion of this program will be presented. The development and evolution of the collaborative partnerships necessary for successful program implementation will be presented in tandem with the usage and evaluation of the programming. Future directions for this work will also be discussed.

